# Waves of Precision: A Practical Guide for Reviewing New Tools to Evaluate Mechanical In-Exsufflation Efficacy in Neuromuscular Disorders

**DOI:** 10.3390/jcm13092643

**Published:** 2024-04-30

**Authors:** Michelle Chatwin, Jesus Sancho, Manel Lujan, Tiina Andersen, Joao-Carlos Winck

**Affiliations:** 1 Neuromuscular Complex Care Centre, The National Hospital for Neurology and Neurosurgery, University College London Hospitals Foundation Trust, London WC1N 3BG, UK; michellechatwin@ymail.com; 2 Clinical and Academic Department of Sleep and Breathing, Royal Brompton Hospital, Part of Guys and St Thomas’ NHS Foundation Trust, London SW3 6NP, UK; 3 Respiratory Medicine Department, Hospital Clínico Universitario, 46010 Valencia, Spain; jesus.sancho@uv.es; 4 Institute of Health Research INCLIVA, 46010 Valencia, Spain; 5 Servei de Pneumologia, Parc Taulí Hospital Universitari, Institut d’Investigació i Innovació Parc Taulí (I3PT-CERCA), Universitat Autònoma de Barcelona, 08208 Sabadell, Spain; mlujan@tauli.cat; 6 Centro de Investigación Biomédica en Red (CIBERES), 28029 Madrid, Spain; 7 Norwegian Advisory Unit on Home Mechanical Ventilation, Thoracic Department, Haukeland University Hospital, 5021 Bergen, Norway; tiina.maarit.andersen@helse-bergen.no; 8 The Faculty of Health and Social Sciences, Western Norway University of Applied Sciences, 5063 Bergen, Norway; 9 Cardiovascular R&D Centre (UniC), Faculdade de Medicina da Universidade do Porto, 4200-319 Porto, Portugal; 10 Pulmonology Unit, Instituto CUF, 4460-188 Porto, Portugal

**Keywords:** mechanical in-exsufflation, peak cough flow, waveform analysis, upper airway obstruction, neuromuscular disorders

## Abstract

Mechanical insufflation-exsufflation (MI-E) is essential for secretion clearance, especially in neuromuscular disorders. For the best outcomes, initiation of MI-E should be started at the correct time with regular evaluation to the response to treatment. Typically, cough peak flow has been used to evaluate cough effectiveness with and without MI-E. This review highlights the limitations of this and discussed other tools to evaluate MI-E efficacy in this rapidly developing field. Such tools include the interpretation of parameters (like pressure, flow and volumes) that derive from the MI-E device and external methods to evaluate upper airway closure. In this review we pinpoint the differences between different devices in the market and discuss new tools to better titrate MI-E and detect pathological responses of the upper airway. We discuss the importance of point of care ultrasound (POCUS), transnasal fiberoptic laryngoscopy and wave form analysis in this setting. To improve clinical practice newer generation MI-E devices should allow real-time evaluation of waveforms and standardize some of the derived parameters.

## 1. Introduction

**Figure 1:**
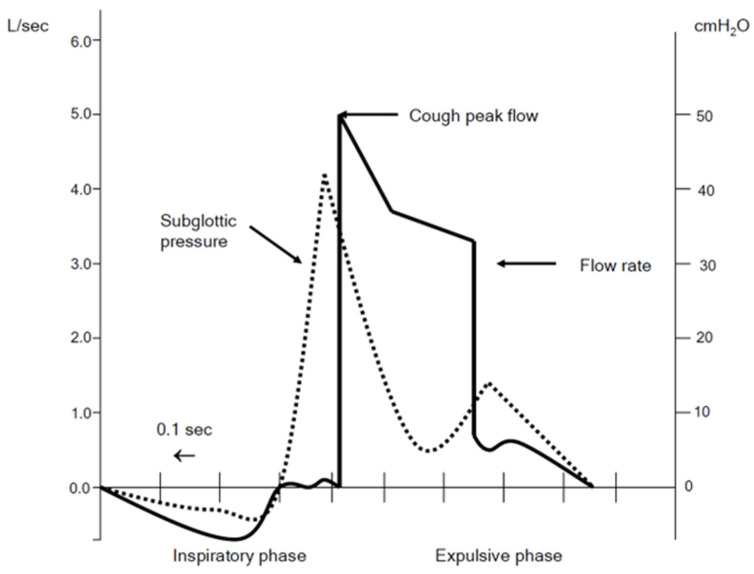
This figure shows the flow changes that occur during a cough in the solid black line. The sub glottic pressure is shown in the dotted line. The inspiratory phase is shown where the flow is negative on the graph. Glottic closure occurs where the flow is zero and where the subglottic pressure becomes positive. The expulsive phase starts as soon as the expiratory flow becomes positive.

An effective cough is a vital mechanism to protect against respiratory tract infections, which are the most common cause of hospital admission in patients with respiratory muscle weakness from neuromuscular disease (NMD) [1,2]. To understand whether MI-E is effective, we first need to understand what a normal cough involves. An effective cough (see Figure 1) has the following three components:

(1) A deep inspiration, up to 85–90% of total lung capacity [3].
(2) Intact bulbar function so that there is rapid closure of the glottis for approximately 0.2 s, coupled with contraction of abdominal and intercostal (expiratory) muscles to generate intrapleural pressures of >190 cm H_2_O [3].
(3) Upon glottic opening, an explosive decompression that generates transient cough peak flows (CPF) [4].

Where there is a suspicion of an ineffective cough, a full clinical and functional assessment should be performed [5]. This involves looking at the inspiratory component, assessing questions like: can the individual take a deep breath in without paradoxical movement? Is there a good expiratory muscle contraction resulting in an audible rapid expulsion of air [6]? When bulbar dysfunction is suspected this can be assessed by asking the patient to repeat “e, e, e, e” to determine whether the vocal cords are opening and closing effectively. The cough expiratory flow can be measured using the cough peak expiratory flow (CPF). This has widely been accepted as a measurement of cough strength. CPF is a simple measurement to perform. It can be measured to obtain a single measurement in time, or it is more useful when measured serially to evaluate improvement or decline in cough strength. CPF measurements should be performed with a full face mask [7] attached to a device that can measure the expiratory flow accurately, either a paediatric peak flow meter (due to it being more accurate at low flows) [8], but may be measured with various devices including portable spirometers or calibrated pneumotachographs. Pneumotachographs have been suggested to be preferable due to their high sampling frequency and greater accuracy [9]. The CPF measurement is the highest expiratory airflow achieved during the cough (see Figure 1). The CPF measurement simply tells us the speed of the expiratory airflow during a cough. In individuals >12 years old, a normal CPF is expected to be greater than 360 L/min [10]. If one or more of the above components are impaired the cough will be less effective [11] and the individual may be unable to produce the transient flow spikes that cause dynamic compression within the flexible airways and are essential for an effective cough [4]. Bach and Saporito evaluated CPF in patients that had an artificial airway removed [12]. From this study, a threshold value of 160 L/min was defined as the level that is needed to be achieved by a cough or cough augmentation technique for an effective cough. In addition to this Tzeng and Bach found that patients who had a CPF of 270 L/min were at risk of deteriorating when unwell to the “critical” level of 160 L/min [13]. As a result of this more recent guidelines recommend the commencement of cough augmentation techniques in patients with a CPF of <270 L/min (DMD and ALS). Further to this, a European Neuromuscular Centre (ENMC) workshop evaluated cough augmentation techniques and classified what techniques are effective at different CPF ranges [14]. This workshop highlighted the fact that, in patients with the lowest CPF (<160 L/min), an MI-E (mechanical insufflation-exsufflation) device (cough machine) is warranted with or without a manual assisted cough. CPF has been widely adopted as the surrogate for cough strength. However, it should not be taken in isolation to determine whether a cough is effective or not or what airway clearance technique including MI-E to start. CPF should be combined with other outcome measures to determine if the cough is effective, and these options are highlighted in Table 1. We all can think of patients who never get respiratory tract infections, who have a low CPF and those that are higher who frequently have respiratory tract infections.

Tabor-Gray and co-workers highlighted that in amyotrophic lateral sclerosis (ALS) patients cough flows looks different to a normal cough. They have prolonged inspiratory phase with reduced peak flow rate and an expiratory rise time is increased with lower peak flows rates [16].

Cough augmentation with MI-E was introduced in the early 1950s and has been shown to have a significant impact in the secretion clearance of patients with neuromuscular disease (NMD) [17,18]. In recent years it has been widely used both in chronic [19] and in acute settings [20], with significant positive clinical outcomes. Different devices have been commercialized [21,22] with diverse characteristics, like the possibility to see or download waveform data. In our review, we provide some guidance on technologies to assist titration and on how to analyze waveform graphics to help clinicians tailor MI-E in the real world.

## 2. Clinical and Functional Options to Evaluate Mechanical Insufflation-Exsufflation (MI-E)

MI-E is a device that delivers positive pressure followed by a rapid switch to negative pressure, thus providing a greater inspiratory volume than the patient can achieve independently. The negative pressure provided by the device helps to increase the expiratory flows that occur naturally when the patient actively coughs. The changes in inspiratory and expiratory flows delivered by MI-E devices aim to simulate the natural flows that occur during a cough. The response of the upper airway to MI-E, both during insufflation and exsufflation, will determine the effectiveness of the technique [23,24]. This response is mainly determined in NMD by bulbar involvement and the type of motor neuron affected, upper or lower [24,25]. This response generates a partial or total obstruction that hinders the passage of air and in turn depends on the pressures applied with MI-E both during insufflation and exsufflation. In this sense, an individualized adjustment of the parameters is nowadays accepted to avoid interference from the upper pathway as much as possible and to achieve an effective technique. We will now describe and discuss options that can assist in evaluating MI-E efficacy.

### 2.1. Cough Peak Flow for the Analysis of MI-E Efficay

**Figure 2:**
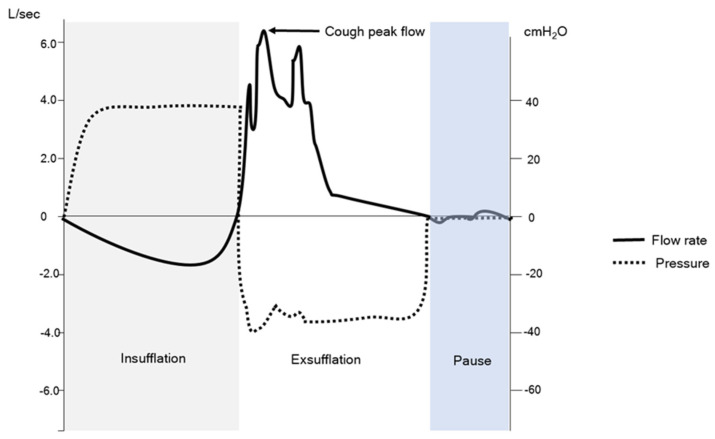
This figure shows a mechanical insufflation exsufflation (MI-E) assisted cough. The insufflation component is in grey, exsufflation in white and the pause in light blue. The cough peak flow (CPF) is also measured as the peak expiratory flow produced with the device. The peak will depend on the sampling time of the device. The pressure swings are shown with the dotted line and the flow changes with the solid line.

**Figure 3:**
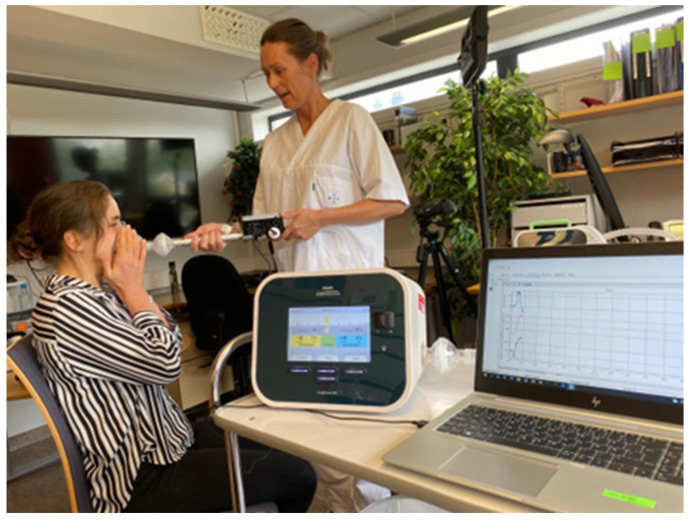
This figure shows the pressure, volume and flow measured by an external gas flow analyzer, Citrex H5 (Regel Medical, GMC Instruments group, Co., Durham, UK). The mask is attached to a metal tubing to assist with laminar flow and accuracy in the reading, then the external gas flow analyzer to the MI-E device 22 mm tubing and then the MI-E device (one option Philips Respironics E70, Murrysville, PA, USA). The external gas flow analyzer is attached to a computer with a digital output. Permission granted to use the person in the pictures image.

An MI-E assisted cough flow trace (See Figure 2) will be different to that of a flow trace seen in an unassisted cough (See Figure 1), but there is also a difference between the flow traces depending on disease [22] and measurement techniques [13,23,24]. MI-E assisted CPF can be measured by the devices internal pneumotachograph. Each company will be accurate at different flow ranges as there are no standard components. An alternative is to use an external device that measures flow and or pressure (Figure 3).

CPF measurements on MI-E devices have their limitations. These are not because each manufacturer measures flows with different components that are not calibrated and therefore give an indication of the MI-E assisted expiratory flow, but when air is sucked out of the device with negative pressure it gives an MI-E assisted CPF reading regardless of what the patient is doing or if the upper airway is patent. Indeed, Terzi and co-workers [21] showed that in a lung model when there was upper airways collapse, the MI-E assisted CPF can be paradoxically high.

Therefore, it is important not to use MI-E assisted CPF alone as the sole evaluation for MI-E efficacy. Other options available on devices can include MI-E volumes, pressure and flow curves.

### 2.2. Mechanical Insufflation-Exsufflation Volumes for the Analysis of MI-E Efficay

Most modern MI-E devices provide more information than the MI-E assisted CPF. Some devices will provide an inspiratory volume and or an expiratory volume. The inspiratory volume on a device will tell us the volume of air not taking into account the possible presence of unintentional leaks. Very low volumes can indicate closure at the laryngeal level due to bulbar dysfunction, or closure due to poor co-ordination by the patient. Sancho and co-workers [25] highlighted the usefulness of inspiratory and expiratory MI-E assisted volumes to detect laryngeal closure. They also highlighted the fact that the inspiratory volume in individuals with upper airway closure the volume was lower than that of an open airway. The changes between an open and an occluded airway were more apparent at higher pressures.

### 2.3. Graphic Analysis, Waveform Evaluation of Cough Mechanics during MI-E for the Analysis of MI-E Efficay

#### 2.3.1. The Normal MI-E Waveform: Variants of Normality; Identification of Points of Interest in Normal Waveforms

The normal morphology of the MI-E curve is not very different from that of a pressure-limited ventilator and the same concepts of interpretation apply to the MI-E curve. However, there are two differences that need to be considered: firstly, the set of a negative pressure during expiration, resulting in a higher-pressure gradient between inspiration and expiration, up to 80 cm H_2_O; and secondly, the maneuver during MI-E is physiologically different from tidal volume ventilation.

As with pressure limited modes, the concept of dependent and independent variables would apply here. The independent variable corresponds to the parameter set in the device (pressure). The dependent variable (flow) reflects the interaction with the device and usually provides more information about the interaction with the patient.

**Figure 4:**
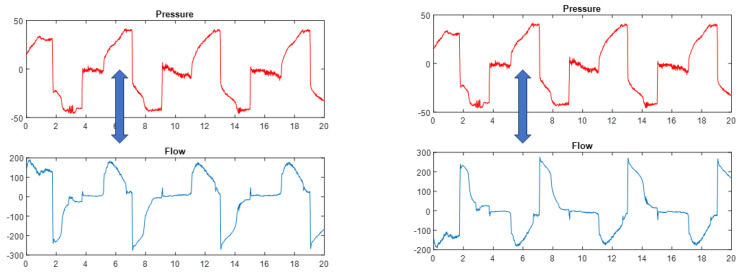
Two different ways of presenting flow waveforms. Right: analogous to NIV; left: with the inverted shape where flow is negative during inspiration and positive during expiration.

Although this may seem a trivial issue, it should be kept in mind that there are two ways of representing the flow waveform. In the first, as in NIV, inspiratory flow is positive and expiratory flow is negative. On the other hand, there are authors who argue that in the case of MI-E, expiratory flow should be recorded as positive as it is the main parameter to be recorded (see Figure 4). The examples in this article will use the NIV-style form, because it seems somewhat counterintuitive to have negative flow when the pressure is becoming positive.

**Figure 5:**
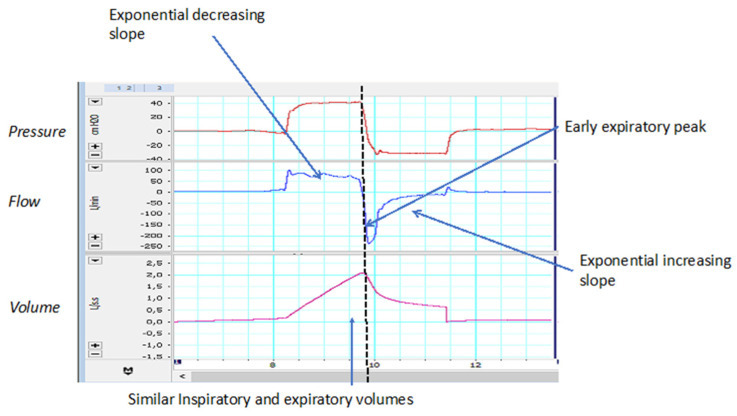
Characteristics of a normal MI-E waveform. See text for more details.

The morphology of the normal inspiratory flow waveform should include an early positive peak and a decreasing exponential morphology after the peak. The expiratory part would include an early negative peak (usually identified with the peak cough flow) and an increasing exponential morphology. Finally, the areas under the curve of inspired and expired flow should show minimal quantitative differences, reflecting no leakage during the performance of the maneuver. In this case, it may be useful to have the volume waveform showing the differences between the inspiratory and expiratory portions (see Figure 5 for details).

**Figure 6:**
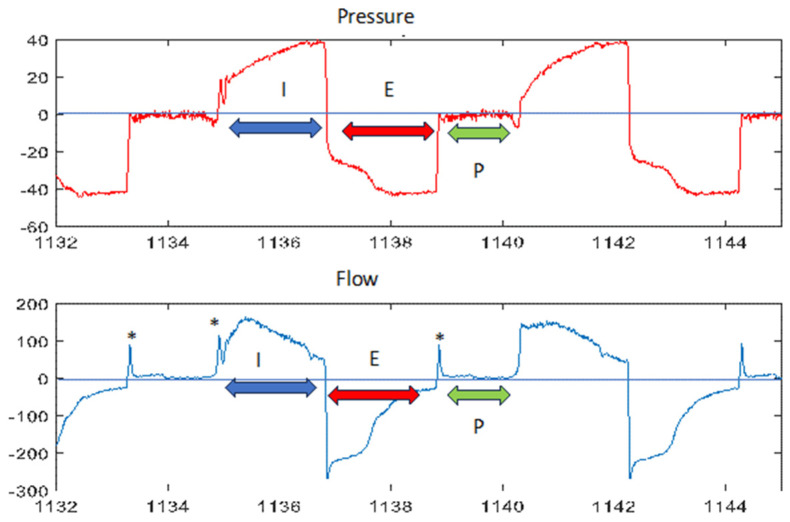
Identification of phases in MI-E. Blue arrow: inspiratory phase. Red arrow: expiratory phase. Green arrow: pause. Asterisk: compressible volume artefacts. See text for more details.

Optionally, a pause can be programmed between exhalation and the next insufflation, corresponding to a value close to zero in both pressure and flow (see Figure 6).

**Figure 7:**
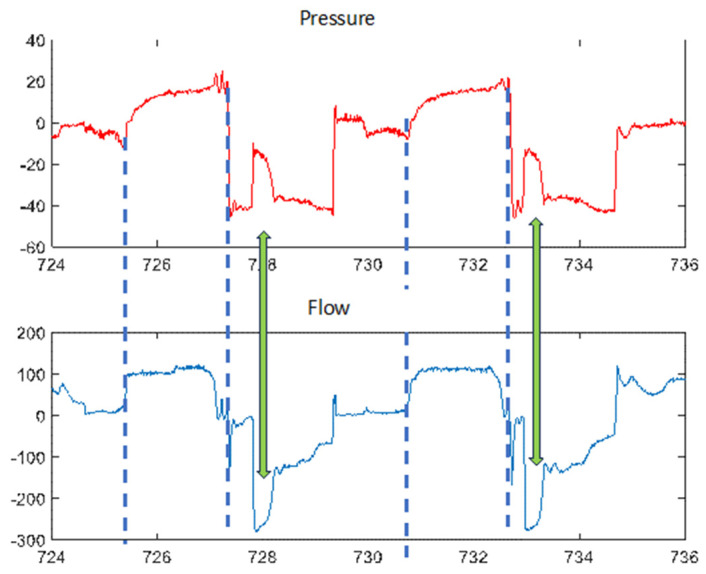
Changes during active cough: more negative peak flow coinciding with less negative peak pressure than in the rest of the phase (green arrow). Inspiratory phase indicated by blue dashed lines. See text for more details.

The morphology of the flow curve may change depending on the active patient’s participation in the procedure. If the patient is instructed to cough during the expiratory phase, it will generate one or more flow peaks of different magnitude and degree of synchronization with the inspiration–expiration transition. Another feature associated with active coughing during the expiratory phase is usually a rise in baseline pressure coinciding with the peak cough, reflecting the inability of the MI-E device to maintain the programmed expiratory pressure in the presence of the patient’s expiratory effort (Figure 7).

An interesting concept that may be misleading in the interpretation of MI-E waveforms is the compressible volume. This phenomenon has also been described in mechanical ventilation [26]. As mentioned above, the pressure gradient between inspiration and expiration is usually higher in MI-E than in NIV. This means that, depending on the compliance of the tubing used, there will be a greater number of gas molecules during inspiration than during expiration. The rapid mobilization of this excess gas causes an early peak flow during the expiratory phase, which is proportional to the pressure difference and should not be confused with the peak cough flow [27]. This peak flow has also been called gas decompression spike [28].

**Figure 8:**
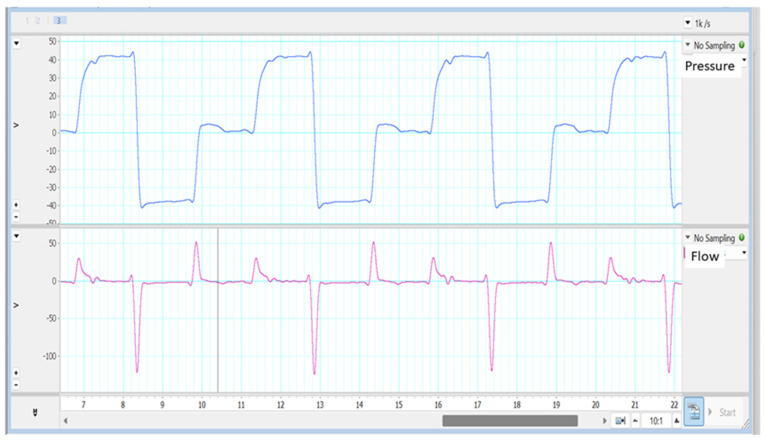
Compressible volume in combination of +40/−40 pressures in MI-E. Note that there is a peak at each transition, but it is maximal when the gradient is maximal (transition from inspiration to expiration).

The most appropriate way to know the magnitude of the compressible volume inside a tubing would be to occlude the circuit at its distal end during an MI-E maneuver (Figure 8). Table 2 reflects the width and height of the compressible volume of a standard 2 m tubing. It should be noted that the mask and the patient’s oral cavity may also be involved in generating the compressible volume. In clinical practice, to avoid confusion with the actual cough peak, all waves with a duration of 100 ms or less should be discarded [27]. Maximum confusion between the actual cough peak and the compressible volume usually occurs during active coughing maneuvers with poor synchronization between the patient and the MI-E device. The compressible volume peak can also be observed at the transition between exhalation and pause, especially at high pressure differences, although in this case the peak will be positive (see Figure 5 and Figure 8).

#### 2.3.2. The Abnormal MI-E Curve

For the classification of abnormalities in the MI-E curve, a system like that proposed by the SOMNO-NIV group for patient-ventilator interactions in non-invasive mechanical ventilation may be useful. This decision algorithm is based on the stratification of abnormalities according to their importance [29,30].

The adaptation of the proposal to MI-E would be as follows.

Major problems: leaks and upper airway obstructions.

**Figure 9:**
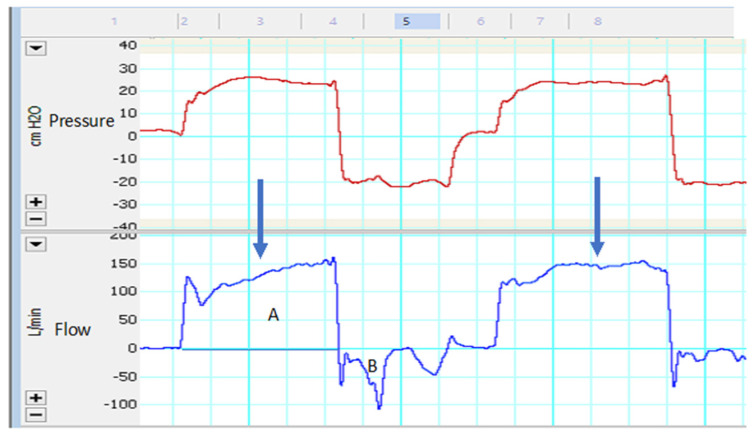
Inspiratory leak tracing. Note the inversion of the flow curve (blue arrow) and the difference between areas A and B of the flow curve.

The presence of leaks in the procedure will distort the flow curve, usually during the inspiratory phase, due to the presence of positive pressure in the circuit. The inspiratory leak will result in a flattening or inversion of the inspiratory curve after the peak flow. Another way to detect inspiratory leaks would be to compare the inspiratory and expiratory portions of the flow curve. Leaks will be suspected if the area under the inspiratory part is clearly larger than the expiratory part (Figure 9).

**Figure 10:**
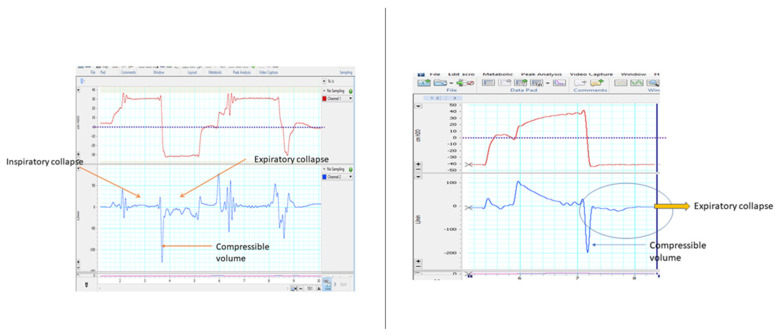
Examples of inspiratory and expiratory collapse (**left**) and expiratory collapse only (**right**). Note that during collapse there is a flattening of the flow curve with values close to zero.

Upper airway narrowing or obstruction is probably one of the most common adverse effects of mechanical assisted cough. Several structures have been described as susceptible to close during mechanical assisted cough [24], but they probably share a common semiology in waveforms. Obstructions will be associated with a flattening of the flow waveform with a value close to zero, both on inspiration and expiration, being a relatively common finding in patients with amyotrophic lateral sclerosis [25]. See examples in Figure 10.

It is important not to confound real obstructions with the transient absence of flow corresponding to the physiological glottic closure phase during MI-E (see below).

Synchronization problems between the patient and MI-E.

**Figure 11:**
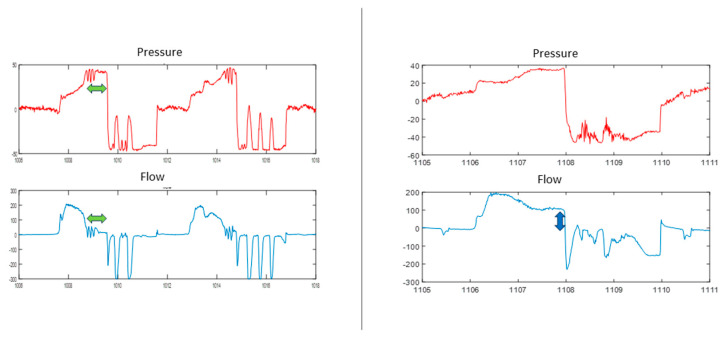
Example of prolonged inspiratory time (**left**) where a plateau occurs at the end of inspiration with values close to zero flow (green arrows); (**right**) shows the opposite situation, i.e., the flow at the time of transition is far from zero, indicating that the situation close to full lung capacity has not yet been reached (blue arrow).

**Figure 12:**
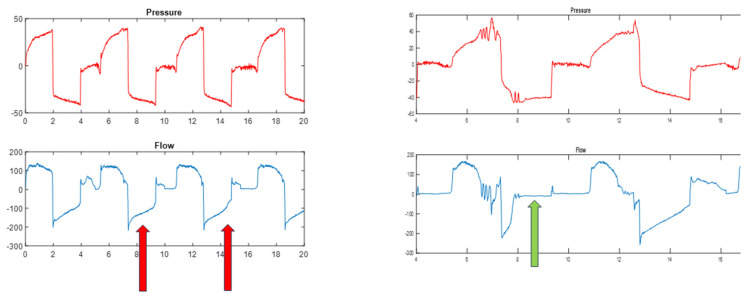
Example of a short expiratory time (**left**), where the negative flow value is still far from the baseline (red arrows), and a prolonged expiratory time (**right**), where after the cough maneuver there is a plateau at the end of the expiratory time with a value close to zero (green arrow).

1. Lack of synchronization between coughing maneuvers and MI-E transition. This is likely to be a very common problem due to the difficulty of synchronizing the patient with the MI-E device in automatic mode and the carer with the patient in manual mode. Active coughing by the patient can occur at any point in the cycle, including inspiration (usually just before the transition to expiration). When it occurs during expiration, it is preceded by a brief period of zero flow, which would correspond to physiological glottic closure.
2. Inspiratory timing discordance. The aim of the inspiratory phase during MI-E is to get as close as possible to total lung capacity. Therefore, if a prolonged plateau at zero flow or close to zero is reached at the end of inspiration, it means that the total lung capacity has been reached earlier and that this excess time at zero flow may be uncomfortable for the patient, resulting in an excessively long inspiratory time. The patient may also react to this excess time with an active exhalation during this inspiratory phase. Conversely, if the patient’s flow rate is well above zero at the transition to exhalation (having ruled out leaks), this may indicate that the inspiratory time can be extended further (see Figure 11).

3. Expiratory timing discordance. The same criteria as in the previous section would apply to the interpretation of the expiratory time: a plateau close to zero flow at the end of expiration would indicate an excessive expiratory time (differential diagnosis with expiratory collapse is mandatory), whereas a flow far from zero at the end of expiration would indicate a short expiratory time (see Figure 12).

Finally, these findings should be integrated into an algorithm for MI-E titration. There are different ways of setting the MI-E parameters. While many studies in North America show good results with fixed pressure combinations, the individualized model is preferred in Europe. Chatwin et al. proposed an algorithm with progressive pressure steps of 5 cm H_2_O starting from a minimum pressure of 20/−20 cm H_2_O [31]. In each of the proposed steps, the clinician can be guided by the information provided by the waveforms: firstly, discarding the cycles with significant leakage; secondly, adjusting pressure levels (reducing pressure if the waveform suggests inspiratory or expiratory closure). Finally, the inspiratory and expiratory times should be adjusted based on the flow levels at the transitions from inspiration to expiration and vice versa.

### 2.4. Auscultation to Detect the Upper Airway and Laryngeal Closure during MI-E, for the Analysis of MI-E Efficay

**Figure 13:**
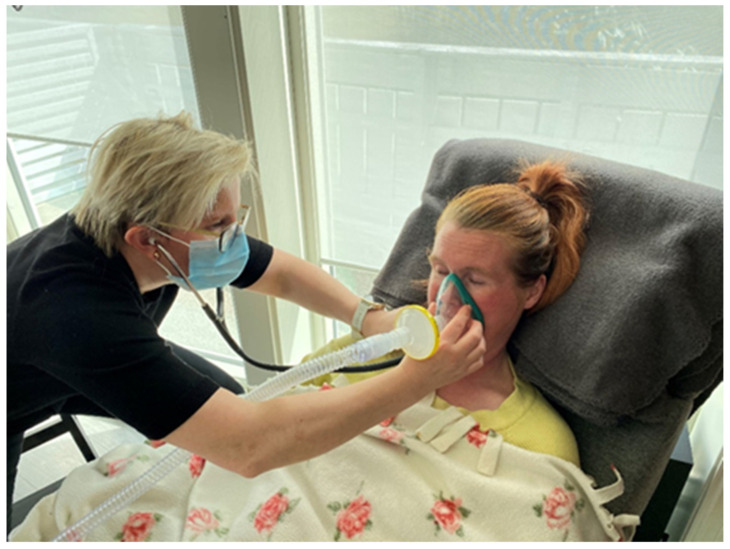
The therapist can listen to the sounds of breathing and the closing of the vocal cords during MI-E therapy by placing a stethoscope on the side of the neck near the larynx. Permission granted to use the persons in the pictures image.

Simple throat auscultation may gain information of laryngeal airflow and synchronization of glottic closure to MI-E cycles, like cervical auscultation used to evaluate swallowing, see Figure 13. You can listen to the sounds of breathing and the closing of the vocal cords during a cough by placing a stethoscope on the side of the neck near the larynx. Listening to these sounds can identify changes airflow during MI-E [32].

### 2.5. Direct Visualization of the Upper Airway, Nasal Fiberoptic Laryngoscopy

Upper airway and laryngeal function play an essential role in the effectiveness of cough as described earlier. When MI-E is administered via a facemask, MI-E pressures and flow need to pass these structures. Some individuals face challenges due to counterproductive upper airway and laryngeal reactions during MI-E, which leads to poor clinical treatment efficacy and compliance. This is especially seen in individuals with bulbar muscular dysfunction, like those with ALS [23,24,25,33]. To understand whether upper airway and laryngeal structures allow the passage of MI-E pressures and flows, firstly, we need to understand what the normal upper airway and laryngeal responses to MI-E are.

**Figure 14:**
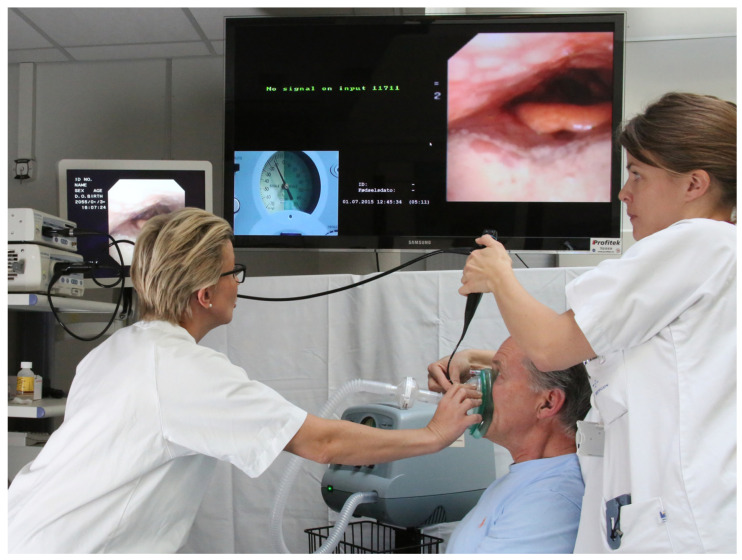
The setup with a laryngoscope passing through a modified interface with the laryngoscope supported and adjusted manually. Situation arranged. Permission granted to use the persons in the pictures image.

Dynamic TFL technique during MI-E (Figure 14) has been useful technique to describe laryngeal response patterns to MI-E both in healthy and in disease [24,33,34,35,36]. In healthy subjects, the larynx acts like a voluntary cough with abducted glottis during both insufflation and exsufflation and exhibits coordinated glottic closure when instructed to cough [34].

In subjects with ALS, regardless of bulbar symptoms, a short initial abduction of the true vocal folds was followed by subsequent adduction, during both insufflation and exsufflation. Subjects with ALS without bulbar symptoms had some difficulties coordinating the laryngeal cough movements during rapid MI-E cycles. A backward movement of the tongue base was prominent during insufflations. Hypopharyngeal constriction during exsufflation was observed in all subjects, regardless of whether they were healthy or had ALS. In subjects with ALS and bulbar symptoms, the laryngeal closure was observed to be prominent during insufflation, especially with high positive pressures. Therefore, prompt, and vigorous delivered insufflation was suggested to be the reason for the failure of MI-E treatment in individuals with bulbar ALS [24]. Andersen and co-workers followed subjects with ALS during the disease progression and observed that the first signs of laryngeal adduction occur at the highest insufflation pressures before any other clinically evident signs of bulbar involvement. Furthermore, clinically MI-E effectiveness decreased, accompanied by laryngeal adduction, even at lower insufflation pressures. Hypopharyngeal constriction during exsufflation appeared later in the disease progression. The positive MI-E pressure could also trigger swallowing reflexes, further complicating the MI-E treatment. Customized use of MI-E, involving lower insufflation pressures and flows over prolonged insufflation time, along with patient-triggered insufflations—resulted in less laryngeal adduction. This approach extended the perceived efficiency of the treatment for subjects with ALS, enabling prolonged and successful use of MI-E [33].

**Figure 15:**
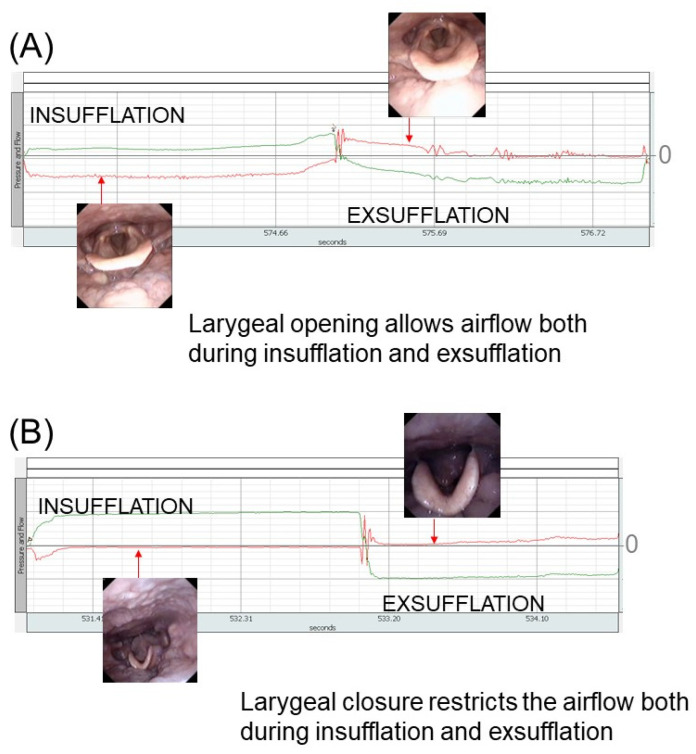
Two examples of MI-E cycles showing airflow and pressure curves during MI-E and a snapshot of the larynx. The pressure swings are shown with the green line and the flow changes with the red line. In example (**A**), the larynx was open both during insufflation and after a glottic closure after the pressure shift from positive to negative. In insufflation, the inspiratory flow fills the lungs, and when switching to the negative pressures, a cough peak flow (CPF) can be detected on expiratory flow tracing. Furthermore, the patient is performing several glottic closures and openings during the exsufflation pressure, and the expiratory flow is declining. In example (**B**), the larynx adducted both during applied insufflation and exsufflation pressures, limiting the airflow significantly.

These inappropriate upper airway and laryngeal responses during MI-E may obstruct the airflow and volumes. This flow structure interaction during MI-E is yet to be labelled systematic. An MI-E assisted cough flow trace with upper airway construction and/or laryngeal closure will be different to that of a flow trace seen with a normal laryngeal cough movement. Figure 15 demonstrates this with two examples.

### 2.6. Computed Tomography to Detect MI-E Efficay

Upper airway computed tomography (CT) scan is an image technique used to value different upper airway pathologies; this technique provides accurate images of the structures of upper airway. A CT scan accurately determines upper cross-sectional area in the supine position and is widely available. Sancho et al. [23], in a study analyzing the efficacy of MI-E in medically stable patients with ALS, performed an upper airway CT scan at baseline and during exsufflation in three patients. The findings show a dynamic collapse of upper airway during exsufflation, with retraction of uvula and reduction of the lateral diameter of pharynx, mainly at the oropharynx, which was greater in those patients with more severe bulbar dysfunction and ineffective mechanically assisted CPF [23]. However, CT scan has disadvantages that limits its use to value response of upper airway to MI-E: radiation exposure, the need to be performed in the Radio diagnostic Department, it is technically difficult to match the triggering of radiation and scanning with insufflation and exsufflation and it is time consuming.

### 2.7. Point of Care Ultrasound (POCUS) to Evaluate the Upper Airway and MI-E Efficay

Direct visualization of upper airway structures and their response by TFL is considered the gold standard [24]. However, TFL has some limitations, including its invasive nature and its availability. In this way, image techniques could be a good alternative to visualize upper airway responses to MI-E.

The use of ultrasound (US) for investigating the upper airway has increased rapidly in different clinical applications with the advantages of availability, lack of radiation, non-invasiveness and real-time imaging. Technical advances with the availability of portable equipment, as well as the growing interest in the technique by different non-radiologist clinicians, has generated the concept of POCUS (US performed and interpreted by the attending clinician where the patient is attended in real time). This represents an important tool for the management of people living with NMD who present a reduced mobility, making it difficult to move around, and significant upper airway responses to MI-E. However, there are currently no published studies on the subject, although there is at least one ongoing study on the subject [37].

In the upper airway US mainly is used the two-dimensional mode with a linear, high frequency (7–15 MHz) transducer, because the majority of the anatomical structures at upper airway are relatively superficial. For the localization of the base of the tongue and oropharynx, and deeper structures, a convex, low frequency (5 MHz) is commonly used. A distance of 3–4 cm is set [38,39]. The structures that can be visualized in the upper airway by US have the following characteristics: bone generates an hyperechoic image, cartilaginous structures are homogeneously hypoechoic, muscle presents as heterogeneous hypoechoic, glandular structures are homogeneous and mildly to strongly hyper-echoic, mucosa-air interface is an hyperechoic line, fluid presents as anechoic and air has the lowest impedance producing both comet tail and reverberation artifacts [38,39].

#### 2.7.1. Ultrasonographic Upper Airway Structures

Fundamentally, the upper airway responses to MI-E occurs at three levels [23,24]: glottis (adduction or paradoxical movement of true vocal folds (TVF) during insufflation), supra-glottis (retroflex movement of epiglottis during insufflation and adduction of aryepiglottic folds during insufflation) and oropharynx (backward movement of the tongue base during insufflation and hypopharyngeal narrowing during exsufflation).

##### Glottis

**Figure 16:**
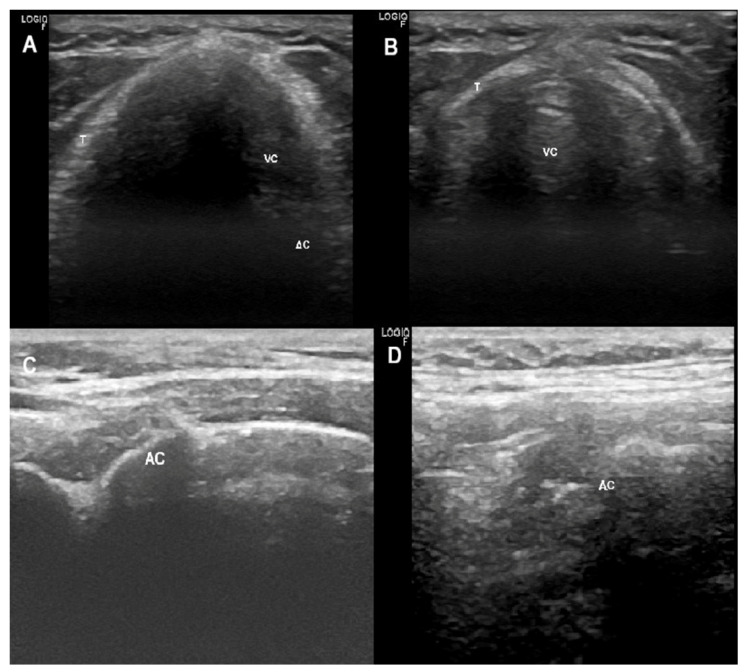
Shows: (**A**) a transverse view at thyroid cartilage using a linear transducer; (**B**) adduction of vocal cords during insufflation; (**C**) a parasagittal view, parallel to the lateral border of thyroid cartilage using a linear transducer; (**D**) adduction of arytenoid cartilage during insufflation. AC = arytenoid cartilage; VC = vocal cords; T = thyroid cartilage.

Thyroid cartilage (TC) represents the best window to visualize glottic structures. The linear transducer is placed at TC in a transverse plane. At this level, TC is visualized as a hypoechoic complete inverted “V”. Deep to TC are TVF which have the appearance of a hypoechoic isosceles triangle that can be delimited medially by the vocal ligaments (VL), hyperechoic lines. The arytenoid cartilages (AC) are deep to the TVF and appear hyperechoic with shadowing (Figure 16A,B).

C movements can also be observed in a parasagittal view, parallel to the lateral border of TC; the transducer is placed at both sides of the TC lamina in order to detect both AC separately (Figure 16C,D).

##### Supra-Glottis

**Figure 17:**
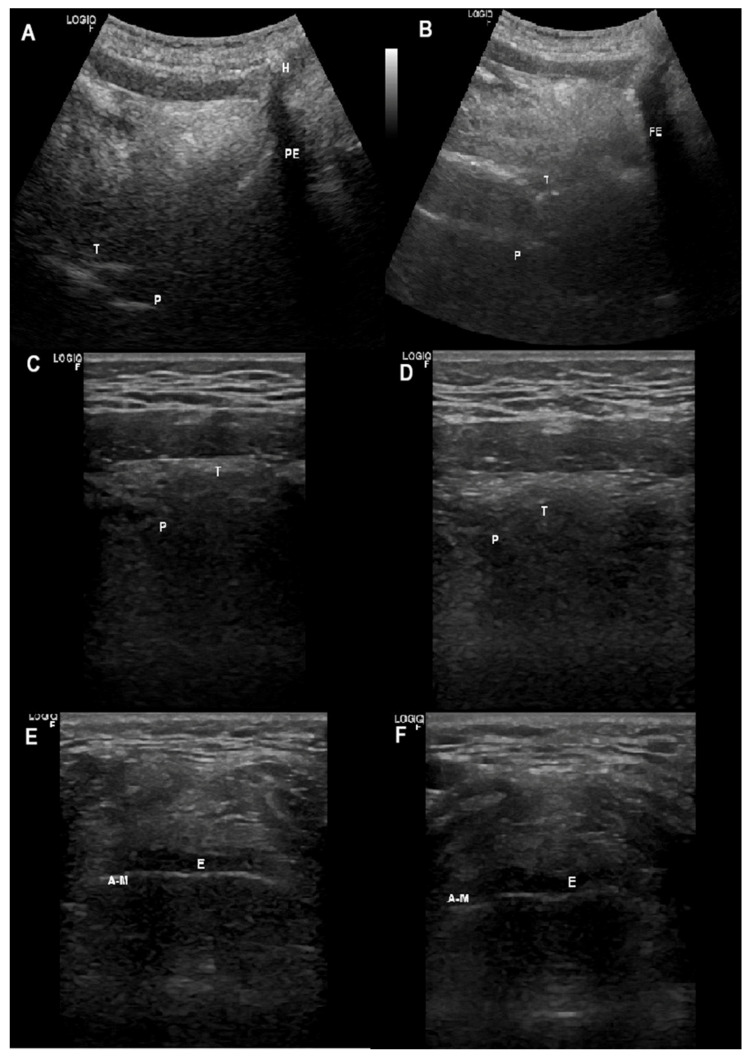
(**A**) Submandibular sagittal view using a curved transducer. (**B**) Backward movement of tongue during insufflation. (**C**) Transverse view in the submandibular position using a linear transducer. (**D**) Oropharyngeal narrowing during exsufflation. (**E**) Transverse view through the thyrohyoid membrane using a linear transducer. (**F**) epiglottis movement during insufflation. A-M = air-mucosa interface; E = epiglottis; H = hyoid bone; P = Palate; PE = pre-epiglottic space; T = tongue.

With the linear transducer in a transverse plane at the thyrohyoid membrane, we can visualize the epiglottis (E). Anteriorly are seen the pre-epiglottic fat and the strap muscles; E appears as a thin hypoechoic stripe lying just anterior to a hyperechoic line representing the air-mucosa interface (A-M) (Figure 17E,F).

##### Oropharynx

To visualize the oropharynx and hypopharyngeal areas, two projections can be used. In the first one, a linear transducer is placed in a transverse plane at submandibular zone, midway between the mentum and hyoid bone. This projection shows the structures in the floor of the mouth. The lingual septum (LS) is visualized as a hyperechoic line in the vertical midline; the tongue is visualized deep to the muscles of the floor of the mouth showing a curvilinear hyperechoic image due to A-M interface. A deeper palate (hypoechoic line) can be detected (Figure 17C,D).

In a sagittal projection, the curve low-frequency transducer is placed submandibular, from the hyoid to the mentum. The tongue is visualized as a curvilineal hyperechoic appearance due to the A-M interface; the palate is localized more deeply (Figure 17A,B).

### 2.8. Other Options to Evaluate MI-E Efficacy

Electrical impedance tomography (EIT) is a functional imaging method that can continuously monitor respiratory function at the bedside. Different measures can be generated from EIT patient examinations allowing the assessment of ventilation distribution, regional lung volume changes and respiratory mechanics, as well as lung perfusion, stroke volume or regional oxygen uptake. EIT has been used to detect changes in ventilation and lung recruitment after MI-E in spinal muscular atrophy type I [40] and DMD [41]. However, if a change (improvement) in ventilation is detected, it can be presumed that the airway is open. Trials are currently taking place to evaluate the effect of MI-E in the ICU, and EIT is one of the methods used to evaluate success [42].

## 3. Future Directions

Further work for the use of POCUS for the efficacy of MI-E is warranted. In addition to this, further studies are required to evaluate whether all patients need to have detailed evaluation of the upper airway response to MI-E or whether this is just for patients with ALS. The availability of equipment and features on MI-E devices often dictates clinical practice. It would be clinically relevant to know what evaluation method the gold standard should be and what is the minimum level of evaluation a patient receives. Perhaps the answer is to partner with industry for the next generation of MI-E devices. This would ensure clinically relevant monitoring and outcome measures should be on a device. In addition, the next generation of MI-E devices should include validated features to help in the detection of upper airway collapse and improve clinical outcomes.

## 4. Conclusions

The use of mechanical in-exsufflation has become more widespread in recent years. Cough effectiveness is commonly evaluated by measuring expiratory CPF. CPF is measured by all new MI-E devices, but its accuracy differs between models. Moreover, using only CPF to ascertain efficacy of cough assistance maybe not be enough. Using other parameters like waveform analysis, TFL or POCUS of the upper airway can be helpful in clinical practice. Prior to utilizing more complex techniques, auscultation of the trachea should not be forgotten as this is a simple method to identify upper airway collapse during MI-E. All these techniques may help personalize this treatment.

## Figures and Tables

**Table 1:** Methods to assess unassisted cough efficacy.

Assessment	Description	Outcome
Patient reported cough efficacy	Patients should be asked whether their cough is effective when well or unwell and if there are any positions the patient clears better with.	Effective or ineffective cough data
Sputum cleared	The ability to clear secretions or not can be used to evaluate cough effectiveness. Either the patient can or cannot clear secretions into the mouth to be cleared independently or orally suctioned from the back of the mouth.	Secretions cleared or retained
Audibility	The auditability of patient’s cough can be used to deem whether it is effective or not. The patient is asked to cough and depending on the strength sound of the cough the cough is deemed as effective or ineffective.	Effective or ineffective cough
Lung function (flow and volume)	Flow volume loops can be performed during a cough. This will give an indication of the inspiratory and expiratory volume as well as the flows achieved. In addition to this transient expiratory flow spikes indicates dynamic compression of the flexible airways.	Production of transient flow spikes indicates an effective cough.
Maximum expiratory pressure (MEP)	This test measures the expiratory muscle strength of the patient.	A MEP > 60 cm H_2_O has been shown to determine an effective cough [6]
Cough peak flow (CPF)	This test will measure the peak expiratory flow during a cough.	A normal cough will generate flows of >360 L/min [10]. An assisted cough that is unable to generate flows < 160 L/min can lead to secretion encumbrance [12].
Cough peak sound via app [15]	This is a smart phone app that is placed close to the patient, and they are asked to cough. It negates the need for a peak flow meter and mask. It estimates the CPF based on the CPS. It has a 94.4% sensitivity and 100% specificity to detect patients with CPF < 270 L/min.	A normal cough will generate flows of >360 L/min [10]. CPF < 270 L/min can identify those at risk of deterioration to a CPF [13].
Auscultation	Auscultation over the trachea can allow the clinician to hear the air flow into the lungs and out. It can be used to determine if there is any upper airway limitation or closure during a spontaneous cough or during mechanical insufflation-exsufflation (MI-E).	Can be used to evaluate whether there is upper airway closure during a cough maneuver.

**Table 2:** Guidelines for peak flow generated by the compressible volume at different pressure combinations in a 2 m standard tubing.

Pressure Difference	Max Negative Flow
40 cm H_2_O (20/−20)	55 L/min
60 cm H_2_O (30/−30)	88.1 L/min
80 cm H_2_O (40/−40)	123.6 L/min

## Data Availability

All data and figures are included in the manuscript.
